# Clinical profile and quality of life in climacteric women

**DOI:** 10.1186/s12912-022-00954-7

**Published:** 2022-08-23

**Authors:** Cíntia Mikaelle Cunha de Santiago Nogueira, Bárbara Daniely dos Santos Silva, Hosana Mirelle Goes Silva Costa, Jussara Rodrigues de Alcantara, Fátima Raquel Rosado Morais, Renata Janice Morais Lima Ferreira Barros, Susy Maria Feitosa de Melo Rabelo, Emanuelly Vieira Pereira, José Rodolfo Lopes de Paiva Cavalcanti, Natália Teixeira Fernandes, Ana Virginia de Melo Fialho

**Affiliations:** 1 Universidade Do Estado Do Rio Grande Do Norte/UERN, Mossoró, RN Brasil; 2 Universidade Estadual Do Ceará/UECE, Fortaleza, CE Brasil

**Keywords:** Quality of life, Climacteric, Menopause

## Abstract

**Background:**

The Quality of Life in climacteric women is intrinsically related to signs and symptoms influenced by the decrease of estrogen and psychosocial factors linked to the natural aging process. Thus, this study aimed to trace the sociodemographic and clinical profiles of climacteric women working at the State University of Rio Grande do Norte (UERN) and evaluate their Quality of Life.

**Methods:**

It is a descriptive, exploratory, and quantitative study. The population consisted of female workers from UERN, aging between 40 and 65 years. The total sample consisted of 63 women who answered the forms. Data collection was completely online, which allowed the women to have free choice to choose the most appropriate time to answer the questions.

**Results:**

The majority of the participants were older than 50 years (53.97%, *n* = 34), were married or in a stable union (55.56%, *n* = 35), and lived in Mossoró-RN (82.53%, *n* = 52). Regarding skin color, white and brown women had the same percentage of 44.44% (*n* = 28). The pain, general health status, and vitality domains evidence the predominance of poor quality of life in the SF-36 questionnaire. Regarding the level of satisfaction with their current health status, 4.76% (*n* = 3) stated that they fit the level considered excellent, 44.44% (*n* = 28) very good, 46, 03% (*n* = 29) were good, and 4.77% (*n* = 3) stated unsatisfied with their current health status. The intensity of symptoms related to estrogen deficiency established by the Kupperman menopause index is as Mild 55.56% (*n* = 35), moderate 41.27% (*n* = 26), and severe 3.17% (*n* = 2).

**Conclusions:**

The occurrence of symptoms and perception of these symptoms differs from woman to woman, to a greater or lesser extent. These findings emphasize the need for qualified and individualized care for the needs of these women in health services and the development of applicable solutions for solving problems based on each woman profile.

## Background

The number of women who go through the climacteric period in Brazil is increasing as the country faces the beginning of a long period of population aging and epidemiological transition. According to studies by the Brazilian Institute of Geography and Statistics [1], the estimative that in 2060 the country will have more elderly than young people.

According to data from the Continuous National Household Sample Survey—Continuous PNAD (2019), which discusses the general characteristics of households and residents, the total population in Brazil is 209.5 million. The number of women is higher than that of men, as it covers 51.8% (108.4 million people) of the total population of Brazil, against 48.2% (101.1 million people) of men. Among the women population in Brazil, 52.9 million range in age from 40 to 64 years, which corresponds to the climacteric period [2].

Another point worth mentioning is that Brazil is experiencing the biggest demographic transition in history as a result of population aging, which has led to a gradual reduction in young people. This point stems from the decrease in fertility and the increase in mortality, which resulted in a change in the population's age, with gradual population aging [2, 3].

Given this evidence, it appears not only that the female population has a high life expectancy but also that there is a tendency to increase and preponderance of this group in the general population since women are in greater numbers and an increasing increase in their life expectancy. Such characteristics confirm the importance of working on health issues linked to aging, such as the climacteric period, since a progressive increase in the demand for health services to deal with complaints related to the theme is expected [4].

In the transitional phase corresponding to the climacteric, usually between 40 and 65 years of age, women undergo several transformations, whether psychosocial, affective, sexual, family-related, or occupational, which can compromise, in a certain way, their quality of life [5]. Climacteric diagnosis is mainly clinical, based on age, changes in the menstrual cycles, and climacteric signs and symptoms [6].

A critical milestone within the climacteric is menopause, which corresponds to the last and definitive menstrual cycle. It can occur between 48 and 50 years of age and is confirmed after 12 consecutive months of amenorrhea [7]. This period is divided into three main phases: pre-menopause, which, in general terms, will designate the period between the beginning of the decline in ovarian function, and can be the period of 3 to 5 years that precedes the last menstruation and in which changes in the menstrual cycle already occur [8]. The second phase is perimenopause, the period close to menopause. Hormonal changes become more intense, generating a shortening or increase in the length of cycles. Finally, there is the post-menopause phase, the period that begins with the last menstruation until the remaining years of a woman's life [8].

In the climacteric period, the production of estrogen and progesterone decreases. The results of these hormonal level changes in women are uncomfortable signs and symptoms such as hot flashes, sweating, irritability, headache, genital atrophy, insomnia, palpitations, dizziness, fatigue, and joint pain [9]. In addition to these physical signs, there are neuropsychic symptoms, such as depression, anxiety, fatigue, and decreased libido [10].

Although the numerous complaints and negative issues attributed to this period, it recognizes that the experience of climacterics differs from woman to woman, deserving comprehensive and differentiated attention in health services [11].

Studies show that the quality of life in climacteric women is intrinsically related to signs and symptoms influenced by estrogen decline and psychosocial factors linked to the natural aging process. In addition to these indicators, social and cultural stigma characterizes the climacteric as a symbol of aging, which can favor feelings of low self-esteem and potentiate other aggravating factors [12].

Thus, it is necessary to measure the quality of life of these women using scales that focus on subjective symptoms and global changes, which allow us to understand how they affect the quality of life, well-being, and relationships in the daily life of these women [13].

Thus, the association of specific scales can make it possible to identify the needs of climacteric women and direct them to specific assistance, which can improve the quality of life of these women [14].

Considering the importance of the topic and the need for holistic nursing care based on the quality of care for climacteric women, a group neglected by the Health System, we aimed to trace the sociodemographic profile, clinical parameters, and quality of life in climacteric women working at the Rio Grande do Norte State University (UERN).

## Methods

This is a descriptive, exploratory, and quantitative study. The population consisted of women working at the Rio Grande do Norte State University aging from 40 to 65 years old.

For better characterization of the sample, we established the following inclusion criteria: women in the pre, peri, and post-menopause period, aging from 40 to 65 years, who agreed to participate in the research after signing the Free and Informed Consent Form. The exclusion criteria were: inability to respond to the interview due to any reason, such as illness and incompatibility of schedules, women with a previous history of bilateral oophorectomy, hysterectomy, and the presence of concomitant and decompensated diseases, such as diabetes mellitus and arterial hypertension.

Data collection occurred online, which allowed the women to have free choice to choose the most appropriate time to answer the questions. The forms were built in Google Forms and sent via email. The first sample consisted of 130 women, among professors, technicians, and deans. The final sample consisted of 63 respondents out of 130.

Four questionnaires were used as data collection instruments to assess the sociodemographic, clinical, and quality of life profile. The first questionnaire analyzed the sociodemographic and socioeconomic characteristics, determined based on the Brazilian Economic Classification criteria of the Brazilian Association of Research Companies. In this system, each item corresponds to a score. The sum of the points gives the rating of the participants in the following classification: class E (up to 4 points), class D (5–9 points), class C (10–20 points), class B (21–34 points), and class A (35 points more).

The second questionnaire analyzed the quality of life of climacteric women and the global changes that can occur in their lives and compromise their quality of life. Such data were measured using the Women's Health Questionnaire, validated in Brazil by Silva Filho [15]. The questionnaire consists of a set of 36 symptoms classified on a four-point scale in descending order and contains nine domains: somatic symptoms, depressed mood, cognitive difficulties, anxiety/fear, sexual functioning, vasomotor symptoms, sleep disturbances, and menstrual symptoms, and whether or not feeling attractive. The higher the score obtained, the more accentuated the dysfunction and the lower the quality of life.

The third questionnaire analyzed general health status, physical and mental components. We use the MOS SF-36 Health Survey, validated in Brazil by Ciconelli [16], to obtain these data. It consists of 36 items into eight domains: functional capacity, physical aspects, pain, general health status, vitality, social and emotional aspects, and mental health.

Finally, the climacteric symptoms were clinically evaluated using the Kupperman Menopausal Index [17], which contains 11 of the most common menopausal complaints. Its totalization occurs through a count that varies from 0 to 51 points. The higher the score, the bigger the level of complaints, and classified as mild, moderate, and severe.

We transferred the data from the Google form to Excel format (2017 version) and used the free statistical software R (version 4.2.0) for descriptive analysis and statistical tests.

Descriptive analysis was performed using absolute and relative frequency distributions (%) to analyze qualitative variables. For the quantitative variables evaluated in the study, we analyzed the following descriptive statistics of trend and data dispersion: minimum, maximum, mean, and standard deviation. To verify data reliability, we used Cronbach's alpha coefficient as a reference, where authors point to data consistency classified as satisfactory for values above 0.70.

To compare data obtained from the sociodemographic profile and from the instruments used in the study, we applied the nonparametric statistical tests of Mann–Whitney, Friedman, and Kruskal Wallis. Spearman test was used for the correlation analyzes of the instruments. In addition, the chi-square test of the general profile of professionals was applied with the classification of the Kupperman menopause index. For all statistical tests, the significance level was 5%.

The Human Research Ethics Committee from Rio Grande do Norte State University, Mossoró, RN, Brazil, approved the study protocol (CAAE n. 38,166,820.6.0000.5294). All the participants signed an informed consent form to participate in this study.

## Results

As shown in Table 1, the majority of participants (53.9%, *n* = 34) ranged in age up to 50 years and 46% (*n* = 29) were older than 50 years old. In addition, 55.5% (*n* = 35) were married or in a stable relationship, 26.9% (*n* = 17) were single and 17.4% (*n* = 11) were divorced. Regarding skin color, whites and browns had the same percentage of 44.4% (*n* = 28), 9.5% (*n* = 6) black and 1.5% (*n* = 1) yellow. As for the city of residence, the majority of women reside in Mossoró (RN), representing 82.5% (*n* = 52).

**Table 1:** Demographic and socioeconomic characteristics

**Characteristics**		**N**	**%**
**Age**	≤ 50 years old	34	53.9
	> 50 years old	29	46
**City of residence**	Mossoró	52	82.5
	Natal	4	6.3
	Pau dos Ferros	2	3.1
	Caicó	1	1.5
	Campina Grande	1	1.5
	Fortaleza	1	1.5
	Portalegre	1	1.5
	Tenente Laurentino Cruz	1	1.5
**Skin color**	White	28	44.4
	Brown	28	44.4
	Black	6	9.5
	Yellow	1	1.5
**Marital status**	Married/Stable union	35	55.5
	Single	17	26.9
	Divorced	11	17.4
**Occupation**	Professor	38	60.3
	Administrative	24	38.1
	Rector/Pro-rector	1	1.5
**Schooling**	PhD	24	38.1
	Master’s degree	20	31.7
	Graduation	16	25.4
	Undergraduation	3	4.7
**Socio-economic status**	A	15	23.8
	B	40	63.4
	C	7	11.1
	D	1	1.5
**Comorbidity**	Yes	27	42.8
	No	36	57.1
**Medication use regularly**	Yes	38	60.3
	No	25	39.6
**Smoking**	Yes	1	1.5
	No	62	98.4
**Regular sports activities**	Yes	38	60.3
	No	25	39.6
**Regular menstrual cycle**	Yes	33	52.3
	No	30	47.6
**Number of pregnancies**	None	10	15.8
	1 or 2	38	60.3
	3 or more	15	23.8
	**Total**	**63**	**100,0**

Source: Research data (2021)

As for their occupation, 60.3% of the participants (*n* = 38) were professors, 38.1% (*n* = 24) were administrative technicians and 1.5% (*n* = 1) rector and pro-rector. Among the participants, 38.1% (*n* = 24) had a PhD degree, 31.7% (*n* = 20) master's degree, 25.4% (*n* = 16) graduate, and 4.7% (*n* = 3) undergraduate degree. Regarding the socio-economic status, the majority of the women in this study (63,4%, *n* = 40) were in class B, 23.8% (*n* = 15) in class A, 11.1% (*n* = 7) and 1.5% (*n* = 1) were in class C and D, respectively.

42.8% of participants (*n* = 27) stated having some concomitant disease, 60.3% (*n* = 38) used medication regularly, 1.5% (*n* = 1) smoked cigarettes, and 60.3% (*n* = 38) practice regular sports activities. 52.3% of women declared having a normal menstrual cycle (*n* = 33) while 47.6% (*n* = 30) did not. Regarding the number of pregnancies, the results were: None (15.8%), 1 or 2 times (60.3%), 3 or more times (23.8%).

The mean length of time of amenorrhea was 6.46 years, as shown in Table 2.

**Table 2:** Descriptive statistics

**Variable**	**Minimum**	**Maximum**	**25%**	**Median**	**75%**	**IQR**	**Mean**	**SD**	**VC**	**p-value**
Age (Years)	40,00	63,00	44,00	49,00	55,00	11,00	49,62	6,23	12,56	0,067
Length of time of amenorrhea (years)	0,33	23,00	2,00	3,00	10,00	8,00	6,46	6,84	105,82	<0,001

Source: Research data (2021)

IQR: interquartile range SD: Standard Deviation CV: Coefficient of Variation (1) Kolmogorov-Smirnov test to verify data normality

As for the SF-36 questionnaire described in Table 3, which represents the level of satisfaction with current health status, 4.7% (*n* = 3) of the respondents stated that they fit the level considered excellent, 44.4% (*n* = 28) very good, 46% (*n* = 29) good and only 4.7% (*n* = 3) poor.

**Table 3:** Current health status satisfaction–SF - 36

** Answer**	**N**	**%**
Excellent	3	4.7
Very good	28	44.4
Good	29	46
Poor	3	4.7
**Total**	**63**	**100.0**

Source: Research data (2021)

The analysis of the variables provided by the WHQ (Table 4) revealed variations between the answers “yes, sometimes” and “yes, without a doubt” for questions related to body pain (*n* = 39, *n* = 7), feeling tired (*n* = 26, *n* = 14), difficulty concentrating (*n* = 25, *n* = 9), tingling (*n* = 22, *n* = 6), loss of sexual interest (*n* = 21, *n* = 5), and headache (*n* = 20, *n* = 4). In addition, other relevant alterations related were poor memory, impaired sleep, feeling of a bloated stomach, irritability, restlessness, and nervousness.

**Table 4:** Frequency distribution of items related to the WHQ instrument

**Item**	**N and %**	**No, ** **not at all**	**No, ** **not much**	**Yes, ** **sometimes**	**Yes, ** **definitely**	**Total**
**I wake early and then sleep badly for the rest of the night**	**n**	13	25	18	7	**63**
	**%**	20.6	39.6	28.5	11.1	**100.0**
**I get very frightened or panic feelings for apparently no reason at all**	**n**	42	14	6	---	**62**
	**%**	67.7	22.5	9.6	---	**100.0**
**I feel miserable and sad**	**n**	33	16	13	---	**62**
	**%**	53.2	25.8	20.9	---	**100.0**
**I feel anxious when I go out of the house on my own**	**N**	47	10	5	---	**62**
	**%**	75.8	16.1	8	---	**100.0**
**I have lost interest in things**	**n**	46	8	7	2	**63**
	**%**	73	12.7	11.1	3.1	**100.0**
**I get palpitations or a sensation of `butterflies in my stomach or chest**	**n**	34	13	15	1	**63**
	**%**	53.9	20.6	23.8	1.5	**100.0**
**I still enjoy the things I used to**	**n**	2	1	16	44	**63**
	**%**	3.1	1.5	25.4	69.8	**100.0**
**I feel life is not worth living**	**n**	49	3	5	4	**61**
	**%**	80.3	4.9	8.2	6.5	**100.0**
**I feel tense or `wound up'**	**n**	21	21	16	4	**62**
	**%**	33.8	33.8	25.8	6.4	**100.0**
**I have a good appetite**	**n**	1	1	14	47	**63**
	**%**	1.5	1.5	22.2	74.6	**100.0**
**I feel restless and can't keep still**	**n**	29	12	15	6	**62**
	**%**	46.7	19.3	24.2	9.6	**100.0**
**I am more irritable than usual**	**n**	15	21	17	9	**62**
	**%**	24.1	33.8	27.4	14.5	**100.0**
**I worry about growing old**	**n**	29	10	14	9	**62**
	**%**	46.7	16.1	22.5	14.5	**100.0**
**I have headaches**	**n**	16	23	20	4	**63**
	**%**	25.4	36.5	31.7	6.3	**100.0**
**I feel more tired than usual**	**n**	12	11	26	14	**63**
	**%**	19	17.4	41.2	22.2	**100.0**
**I have dizzy spells**	**n**	30	16	15	1	**62**
	**%**	48.3	25.8	24.1	1.6	**100.0**
**My breasts feel tender or uncomfortable**	**n**	31	17	12	2	**62**
	**%**	50.0	27.4	19.3	3.2	**100.0**
**I suffer from backache or pain in my limbs**	**n**	7	10	39	7	**63**
	**%**	11.1	15.8	61.9	11.1	**100.0**
**I have hot flushes**	**n**	29	15	10	9	**63**
	**%**	46	23.8	15.8	14.2	**100.0**
**I am more clumsy than usual**	**n**	28	13	13	7	**61**
	**%**	45.9	21.3	21.3	11.48	**100.0**
**I feel rather lively and excitable**	**n**	7	11	30	13	**61**
	**%**	11.4	18.0	49.1	21.3	**100.0**
**I have abdominal cramps or discomfort**	**n**	27	21	11	3	**62**
	**%**	43.5	33.8	17.7	4.8	**100.0**
**I feel sick or nauseous**	**n**	43	10	8	---	**61**
	**%**	70.5	16.3	13.1	---	**100.0**
**I have lost interest in sexual activity**	**n**	21	14	21	5	**61**
	**%**	34.4	22.9	34.4	8.2	**100.0**
**I have feelings of well-being**	**n**	2	5	25	30	**62**
	**%**	3.2	8	40.3	48.3	**100.0**
**I have heavy periods **	**n**	32	8	12	8	**60**
	**%**	53.3	13.3	20	13.3	**100.0**
**I suffer from night sweats**	**n**	34	13	9	6	**62**
	**%**	54.8	20.9	14.5	9.6	**100.0**
**My stomach feels bloated**	**n**	18	15	18	10	**61**
	**%**	29.5	24.5	29.5	16.3	**100.0**
**I have difficulty in getting off to sleep**	**n**	21	17	16	9	**63**
	**%**	33.3	26.9	25.4	14.2	**100.0**
**I often notice pins and needles in my hands and feet**	**n**	26	9	22	6	**63**
	**%**	41.2	14.2	34.9	9.5	**100.0**
**I am satisfied with my current sexual relationship (please omit if not sexually active)**	**n**	4	8	21	17	**50**
	**%**	8	16	42	34	**100.0**
**I feel physically attractive**	**n**	4	8	32	18	**62**
	**%**	6.4	12.9	51.6	29	**100.0**
**I have difficulty in concentrating**	**n**	13	16	25	9	**63**
	**%**	20.6	25.4	39.6	14.2	**100.0**
**As a result of vaginal dryness sexual intercourse has become uncomfortable (please omit if not sexually active)**	**n**	26	8	9	7	**50**
	**%**	52	16	18	14	**100**
**I need to pass urine/water more frequently than usual**	**n**	29	10	16	7	**62**
	**%**	46.7	16.1	25.8	11.2	**100.0**
**My memory is poor**	**n**	17	18	17	11	**63**
	**%**	26.9	28.5	26.9	17.4	**100.0**

Source: Research data (2021)

According to Table 5, among the clinical signs evaluated as severe, the predominance of responses refers to insomnia (17.4%, *n* = 11). The main symptoms considered moderate were arthralgia/myalgia (36.5%, *n* = 23), followed by signs of headache (33.3%, *n* = 21). Regarding the mild symptoms, the predominance refers to vasomotor symptoms (52.3%, *n* = 33) and signs of nervousness (49.2%, (*n* = 31).

**Table 5:** Frequency distribution of items related to the Kupperman index instrument

**Items**	**N and %**	**None**	**Mild**	**Moderate**	**Severe**	**Total**
**Vasomotor symptoms**	**n**	21	33	6	3	**63**
	**%**	33.3	52.3	9.5	4.7	**100.0**
**Paresthesia**	**n**	20	28	11	4	**63**
	**%**	31.7	44.4	17.4	6.3	**100.0**
**Insomnia**	**n**	18	22	12	11	**63**
	**%**	28.5	34.9	19	17.4	**100.0**
**Nervousness**	**n**	13	31	17	2	**63**
	**%**	20.6	49.2	26.9	3.1	**100.0**
**Melancholia**	**n**	22	27	13	1	**63**
	**%**	34.9	42.8	20.6	1.5	**100.0**
**Vertigo**	**n**	20	30	12	1	**63**
	**%**	31.7	47.6	19	1.5	**100.0**
**Weakness/Fatigue**	**n**	23	29	10	1	**63**
	**%**	36.5	46	15.8	1.5	**100.0**
**Arthralgia or myalgia**	**n**	12	22	23	6	**63**
	**%**	19	34.9	36.5	9.5	**100.0**
**Headaches**	**n**	13	24	21	5	**63**
	**%**	20.6	38.1	33.3	7.9	**100.0**
**Palpitation**	**n**	24	28	11	---	**63**
	**%**	38.1	44.4	17.4	---	**100.0**
**Formication**	**n**	18	27	12	6	**63**
	**%**	28.5	42.8	19	9.5	**100.0**

Source: Research data (2021)

In an analysis of Table 6, women aged up to 50 years had higher SF36 scores for pain and higher WHQ scores for menstrual problems, in addition to lower WHQ scores for vasomotor symptoms and a lower Kupperman menopausal index. Married professionals or those in a stable relationship presented higher WHQ scores on somatic symptoms and sexual and global functioning on the WHQ, in addition to lower SF-36 scores on vitality.

**Table 6:** Comparison between the scales used in the study and the age of the professionals

**Domains**	**Age**	**Minimum**	**Maximum**	**25%**	**Median**	**75%**	**IQR**	**Mean**	**SD**	**CV**	**p-value**
**Functional capacity**	**≤ 50 years**	30,00	100,00	70,00	90,00	100,00	30,00	82,79	18,76	22,65	**0,052**
	**> 50 years**	35,00	100,00	55,00	75,00	95,00	40,00	73,28	21,22	28,97	
**Limitation due to physical aspects**	**≤ 50 years**	0,00	100,00	50,00	100,00	100,00	50,00	75,74	33,98	44,87	**0,217**
	**> 50 years**	0,00	100,00	25,00	75,00	100,00	75,00	64,66	38,68	59,83	
**Pain**	**≤ 50 years**	31,00	100,00	62,00	72,00	84,00	22,00	70,79	19,66	27,77	**0,028**
	**> 50 years**	0,00	100,00	41,00	62,00	72,00	31,00	57,69	24,09	41,76	
**General health status**	**≤ 50 years**	12,00	80,00	42,00	47,00	57,00	15,00	48,91	12,25	25,04	**0,300**
	**> 50 years**	22,00	82,00	42,00	52,00	62,00	20,00	52,45	14,56	27,75	
**Vitality**	**≤ 50 years**	20,00	75,00	55,00	60,00	65,00	10,00	55,59	13,86	24,93	**0,397**
	**> 50 years**	30,00	80,00	55,00	60,00	65,00	10,00	58,79	10,91	18,56	
**Social aspects**	**≤ 50 years**	25,00	100,00	50,00	75,00	87,50	37,50	71,69	22,04	30,75	**0,669**
	**> 50 years**	25,00	100,00	50,00	75,00	100,00	50,00	72,84	25,68	35,25	
**Limitation by emotional aspects**	**≤ 50 years**	0,00	100,00	33,33	100,00	100,00	66,67	67,65	40,61	60,03	**0,982**
	**> 50 years**	0,00	100,00	33,33	100,00	100,00	66,67	67,82	39,32	57,98	
**Mental health**	**≤ 50 years**	36,00	92,00	64,00	74,00	84,00	20,00	73,29	13,95	19,03	**0,814**
	**> 50 years**	44,00	96,00	56,00	76,00	88,00	32,00	71,31	17,63	24,73	
**Somatic symptoms**	**≤ 50 years**	28,57	89,29	39,29	50,00	60,71	21,43	50,53	13,68	27,08	**0,186**
	**> 50 years**	25,00	82,14	46,43	53,57	67,86	21,43	55,91	16,62	29,73	
**Depressed mood**	**≤ 50 years**	25,00	66,67	29,17	39,58	50,00	20,83	41,30	11,93	28,90	**0,536**
	**> 50 years**	20,83	66,67	29,17	37,50	45,83	16,67	39,37	11,76	29,88	
**Cognitive difficulties**	**≤ 50 years**	25,00	100,00	41,67	50,00	75,00	33,33	56,13	23,78	42,36	**0,808**
	**> 50 years**	16,67	100,00	41,67	58,33	66,67	25,00	56,32	22,01	39,08	
**Anxiety**	**≤ 50 years**	25,00	81,25	25,00	37,50	43,75	18,75	39,52	14,33	36,25	**0,695**
	**> 50 years**	18,75	75,00	25,00	37,50	50,00	25,00	41,16	15,45	37,53	
**Sexual functioning**	**≤ 50 years**	8,33	100,00	25,00	37,50	58,33	33,33	42,65	21,00	49,24	**0,427**
	**> 50 years**	8,33	83,33	25,00	50,00	66,67	41,67	47,53	24,22	50,96	
**Vasomotor symptoms**	**≤ 50 years**	12,50	100,00	25,00	25,00	37,50	12,50	36,76	20,63	56,11	**<0,001**
	**> 50 years**	25,00	100,00	37,50	50,00	75,00	37,50	58,62	25,68	43,81	
**Sleep problems**	**≤ 50 years**	25,00	100,00	33,33	50,00	58,33	25,00	50,00	19,03	38,05	**0,081**
	**> 50 years**	25,00	100,00	50,00	50,00	75,00	25,00	58,05	20,35	35,06	
**Menstrual problems**	**≤ 50 years**	25,00	100,00	37,50	53,13	68,75	31,25	53,49	18,09	33,81	**0,006**
	**> 50 years**	12,50	87,50	25,00	37,50	50,00	25,00	40,95	17,80	43,48	
**Attraction**	**≤ 50 years**	25,00	83,33	41,67	41,67	58,33	16,67	49,26	15,40	31,26	**0,368**
	**> 50 years**	33,33	83,33	41,67	50,00	58,33	16,67	52,30	13,34	25,51	
**WHQ**	**≤ 50 years**	31,94	69,44	37,50	45,83	55,56	18,06	46,43	10,67	22,99	**0,460**
	**> 50 years**	26,39	77,78	41,67	46,53	56,25	14,58	48,47	12,21	25,19	
**Kupperman index**	**≤ 50 years**	0,00	35,00	5,00	13,50	21,00	16,00	14,21	10,30	72,49	**0,012**
	**> 50 years**	4,00	39,00	15,00	20,00	29,00	14,00	20,97	9,82	46,85	

Source: Research data (2021)

IQR: interquartile range SD: Standard Deviation CV: Coefficient of Variation

Professionals with a regular menstrual cycle had higher SF-36 scores for functional capacity and WHQ scores for menstrual problems and lower WHQ scores for vasomotor symptoms. Women with a concomitant disease had higher scores in the respective dimensions mentioned. Professionals who practice regular physical activity had higher scores in the mentioned dimension.

Professionals who had three or more pregnancies scored higher on sleep problems, somatic symptoms, and the Kupperman menopause index, while professionals with 1 or 2 pregnancies had higher pain scores.

In Table 7, using the general Kupperman score, there is the following classification for the situation of the occurrence of clinical signs in the climacteric: Mild 55.5% (*n* = 35), moderate 41.2% (*n* = 26), and severe 3.1% (*n* = 35). = 2).

**Table 7:** Kupperman menopausal index rating

**Evaluation**	**N**	**%**
Mild	35	55.5
Moderate	26	41.2
Severe	2	3.1
**Total**	**63**	**100.0**

Source: Research data (2021)

## Discussion

In this study, the predominance of poor quality of life in the SF-36 was evidenced in the domains referring to pain, general health status, and vitality. In this sense, it is worth mentioning that other studies have identified a compromise in the vitality domain, possibly due to the working hours that women perform inside and outside their homes [18].

For the most part, when asked about the level of satisfaction with their health, women responded that they were either good or very good, and only a portion referred to their health as poor. However, this finding can be justified, in part, by the economic classification and levels of education they belong to, which, being more favorable, provide opportunities for better dietary practices, physical activities, and other measures that promote healthy habits [19]. On this issue, a study from Brazil showed that schooling was one of the predominant factors for better coping with the climacteric period since it provides knowledge about its difficulties, symptoms, and, mainly, forms of treatment and alleviation of symptoms [20].

In addition, the economic level is another fundamental factor for the improvement of the quality of life of these women because, given the social and health situation in the country, it provides access to specialized care services for their climacteric complaints, with a more professional, qualified, and multi-professional service. Thus, humanized, prepared, and informative service has a positive effect on the perception of these women's quality of life [6].

Furthermore, studies on climacteric women have shown that regular physical activity is another factor that positively contributes to a lower manifestation of symptoms and improves the quality of life [21]. Practices are responsible, for example, for improving mood and relieving hot flashes and, therefore, contribute to a better perception of quality of life [22]. The predominant factor in this study was the large portion (60.32%) of women who participated in this research performed physical activities regularly.

The pain was one of the SF-36 domains that also varied, which may be associated with the work carried out, physical effort, and issues of non-ergonomics in the work environment [23]. In addition, other studies relate pain to hormonal changes, especially hypoestrogenism, which is associated with bone cartilage wear [24]. In addition, musculoskeletal pain was the most frequent complaint, affecting about 93% of the population studied. The majority classified the pain as intense [25].

The WHQ domains that showed more impairments were related to depressed mood, anxiety, sexual functioning, vasomotor symptoms, and menstrual problems. About 50 to 70% of women who experience the climacteric period can face emotional problems, loss of libido, anxiety, and even depression. Furthermore, this author also points to the relationship between depression, mainly associated with the fear of aging, and the feeling of uselessness and affective lack [26].

Studies indicate that between 25 and 35% of women from 35 to 59 years old tend to have sexual dysfunctions, reaching up to 75% among women aged 60 to 65. This dysfunction is evidenced by the urogenital atrophy mechanism but mainly by the physical changes that occur with aging, impacting self-esteem and libido [20]. In line with these findings, studies conducted in Sweden showed that most women reported sexual dysfunctions, such as decreased libido, sexual activity, satisfaction, and symptoms associated with vaginal dryness [27].

Another problem that compromises the quality of life of the analyzed women was the somatic symptoms domain, characterized by hot flushes and sweating. About 75% of menopausal women states having hot flushes [28]. Such symptoms tend to compromise the quality of life of these women. In studies developed, they showed a small incidence of hot flushes in pre-menopause, with an increase in these symptoms in early perimenopause and a higher incidence in late perimenopause, however, after menopause, especially in older women, there is a decline in the intensity of these symptoms [29].

The Kruskal-Walli’s test showed a statistical difference between the number of pregnancies and the SF-36 pain domain, sleep problems, and WHQ somatic symptoms. This finding corroborates other studies in which the association between more pregnancies and the intensity of climacteric symptoms, shows that women with three children or more had more severe menopausal symptoms [30].

There were no statistical differences between the quality of life and smoking, probably due to the low number of female smokers. However, studies show an association between a poor quality of life and the habit of smoking [31].

However, there is a statistical difference between marital status and the SF-36 vitality domain, somatic symptoms, and sexual and global functioning from the WHQ. Married professionals or those in a stable relationship scored higher on WHQ somatic symptoms and sexual and global functioning domains, in addition to a lower SF-36 score on vitality. These findings corroborate other studies that point to a correlation between a better quality of life in women living with a partner compared to those who declare themselves single or divorced [32].

We found a statistical difference between the presence of concomitant disease and the WHQ and Kupperman Menopause Index anxiety domain. Professionals with a concomitant disease scored higher in the dimensions mentioned. The chance of professionals with the concomitant disease to present moderate or severe Kupperman classification increases 5.20 × compared to professionals without concomitant disease [33].

Finally, the chance of professionals using regular medication to present moderate or severe Kupperman classification increases 4.35 × compared to those who do not use medication regularly. These corroborate a study conducted in the city of Ouro Preto with approximately 113 climacteric women, which verified that the presence of chronic diseases and use of medications along with the climacteric was associated with a worse quality of life [33].

The intensity of symptoms related to estrogen deficiency established by the Kupperman menopause index was classified as Mild 55.56% (*n*° = 35), moderate 41.27% (*n*° = 26), and severe 3.17% (*n*° = 2). Insomnia symptoms were perceived as the most marked change, while symptoms related to arthralgia/myalgia and headache were considered moderate.

Insomnia problems are common in the climacteric period. However, there are no studies correlating insomnia and estrogenic decrease. This domain is more often associated with hot flushes and emotional difficulties [26]. There is a positive relationship between the sleep scale and the score related to menopause, which indicates a worse sleep quality in women experiencing this period [34]. Thus, insomnia, along with hot flushes, are the main complaints of women during menopause [28]. Studies indicate that the use of sleeping pills increases from 5.8% to 11.22% in the female population who are going through menopause [35].

Therefore, the results found in this research corroborate with several studies on the climacteric theme that mention the arrival of this period in a woman's life being marked by several changes, whether physical, hormonal, or emotional. The occurrence of symptoms and perception of these symptoms differs from woman to woman, to a greater or lesser extent. These emphasize the need for qualified and individualized care for the needs of these women in health services.

## Conclusions

This study showed that the occurrence of climacteric signs and symptoms are mainly associated with basal hormone levels, but, above all, with the way women experience this stage of life, with all the physical and emotional changes. Adopting measures that promote a better quality of life and self-esteem, such as healthy habits, adequate nutrition, regular physical activities, and relaxing moments can provide better health and improve quality of life.

Thus, it corroborates with other authors when stating that quality of life is associated with signs and symptoms and with the way of seeing and facing life, reality, and adversities [6]. Thus, health professionals, especially nurses, should develop actions that promote the quality of life in climacteric women by focusing on well-being, self-care, self-esteem, and complication prevention.

## Data Availability

The data that support the findings of this study are available from the corresponding author upon reasonable request.
